# Supplementary material for: Agricultural education in Africa using YouTube multilingual animations: A retrospective feasibility study assessing costs to reach language-diverse populations
Source: PLoS One. 2024 Apr 18;19(4):e0302136. doi: 10.1371/journal.pone.0302136 (PMC11025858; doi:10.1371/journal.pone.0302136)
Supplement: S1 File — Links to YubeTube multilingual animations and geographical locations targeted in YouTube Ad campaigns. (DOCX) [file pone.0302136.s001.docx]

**S1 YouTube Ad campaigns**

YouTube ad campaigns were targeted by language and location. Please refer to Table S1.1 for information related to the Ghanian campaigns, Table S1.2 for the Kenyan campaigns, and Table S1.3 for the Nigerian campaigns.

*Table S1.1: Jerrycan YouTube ad campaigns in Ghana.*

| Language | YouTube Link | Location specified in YouTube Ad Campaign |
| --- | --- | --- |
| Akuapem | https://youtu.be/ge7ViZGQk24 | Eastern Ghana |
| Asante Twi | <https://youtu.be/zmbP6MuLQnk> | Ashanti, Bono, Bono east, Ahafo |
| Bono | <https://youtu.be/5hE24a0MXI0> | Country wide |
| Dagaare | <https://youtu.be/J7VOuf7B05E> | Country wide |
| Dagbani | <https://youtu.be/uc0oYL04KWk> | Northern, Savannah |
| Dangme | <https://youtu.be/0EmqNfE_zdg> | Northern, Savannah |
| English | <https://youtu.be/9Hc2TE_SA1A> | Country Wide |
| English | <https://youtu.be/9Hc2TE_SA1A> | Ghana ZOI |
| Ewe | <https://youtu.be/pml6tp8hXbI> | Volta region, Oti |
| Fante | <https://youtu.be/cPHjMaZZtk0> | Central, Western Region, Western North |
| Farefare | <https://youtu.be/NrHrkl5JCSs> | Upper East |
| Ga | <https://youtu.be/QljdNCpcirI> | Greater Accra |
| Gonja | <https://youtu.be/NZOo1kPZVKk> | Northern, Savannah |
| Kasem | <https://youtu.be/GhOvwma2-K0> | Upper East |
| Kusaal | <https://youtu.be/qoUUkYPLaAQ> | Upper East |
| Nzema | <https://youtu.be/W8bLBvzAIlk> | Western region |
| Sisaali | <https://youtu.be/nSaV93Tsr0Q> | Country wide |

Ghana ZOI represents the following locations in Ghana: Eastern Ghana, Ashanti, Bono, Bono East, Northern, Savannah, Volta region, Oti, Central, Western Region, Western North, Upper East, and Greater Accra.*Table S1.2 Jerrycan YouTube ad campaigns in Nigeria.*

| Language | YouTube Link | Location specified in YouTube Ad Campaign |
| --- | --- | --- |
| Abi | <https://youtu.be/0bL6iddu9AI> | Country wide |
| Bura-Pabir | <https://youtu.be/cBhdtyftPbk> | Borno state, Adamawa state, Borno state, Taraba state, Yobe state |
| Edo | <https://youtu.be/lf3wUtCmzXI> | Edo state |
| Efik | <https://youtu.be/nJ20uSEF9TU> | Cross River |
| English | <https://youtu.be/BZtUSs4vEcc> | Country wide |
| English | <https://youtu.be/BZtUSs4vEcc> | ZOI |
| Fulfulde | <https://youtu.be/2xio0QmhZGI> | Adamawa state |
| Hausa | <https://youtu.be/z_R5HGZiiuE> | Benue, Kaduna, Kebbi, Kebbi, Niger |
| Igbo | <https://youtu.be/0SM7bcDv8O4> | Ebonyi |
| Ijaw | <https://youtu.be/fsQFxv4ZXCY> | Delta, |
| Isoko | <https://youtu.be/J7mQWybIwcY> | Country wide |
| Itshekiri | <https://youtu.be/SZeWu-CVOO8> | Country wide |
| Kanuri | <https://youtu.be/TAJweIMooyo> | Borno State |
| Kalabari | <https://youtu.be/26rCY2Q6HCk> | Country wide |
| Karekare | <https://youtu.be/ubIaBUlx0Bs> | Yobe State and Bauchi |
| Marghi | <https://youtu.be/kRgOcqdDt-k> | Adamawa state, Borno state, Gombe state, Taraba state and Yobe state |
| Nupe | <https://youtu.be/GwTEKNIQtTY> | Niger state |
| Pidgin | <https://youtu.be/6ioxTwfd0DY> | Benue, Cross River, Delta, Ebonyi, Kaduna, Kebbi, Niger |
| Tangale | <https://youtu.be/mPFcpHMqxlY> | Gombe state, Bauchi state |
| Tiv | <https://youtu.be/_0YztcBL_nQ> | Benue |
| Urhobo | <https://youtu.be/imOwsIRTPXo> | Country wide |
| Yakkur | <https://youtu.be/Rqqd80VpFDk> | Country wide |
| Yoruba | <https://youtu.be/XOf8oPvbO6M> | Ogun State, Oyo State, Lagos State, Ekiti State, Ondo State and Osun State |

Nigeria ZOI represents the following locations in Nigeria: Benue, Cross River, Delta, Ebonyi, Kaduna, Kebbi and Niger.

*Table S1.3: Jerrycan YouTube ad campaigns in Kenya.*

| Language | YouTube Link | Location specified in YouTube Ad Campaign |
| --- | --- | --- |
| Bukusu | <https://youtu.be/6qdofMf9mM0> | Bungoma, Busia, Transnzoia |
| Chidigo | <https://youtu.be/v9B4HD3dAMQ> | Country wide |
| Chonyi | <https://youtu.be/EIcw-7s_6LU> | Kilifi County |
| Dawida | <https://youtu.be/PRJHobb1QFU> | Taita Taveta |
| Duruma | <https://youtu.be/gtDAqirICwM> | Kilifi county, Kwale county |
| Embu | <https://youtu.be/oeo4jUQ_vfQ> | Embu county |
| English | <https://youtu.be/lymS2eVdeUQ> | Country wide |
| English | <https://youtu.be/lymS2eVdeUQ> | Kenya ZOI |
| Giryama | <https://youtu.be/ILcamDmWofY> | Kilifi county, Mombasa, Kwale, Lamu county |
| Gusii | <https://youtu.be/n751kZbpimE> | Kisii, Nyamira, Migori counties |
| Kamba | <https://youtu.be/Q09KzXixGtg> | Kitui, Machakos, Makueni |
| Kidigo | <https://youtu.be/NW6NXxMJj-U> | Kwale, Kilifi, Mombasa, Lamu counties |
| Kikuyu | <https://youtu.be/BuxYZFpk5oY> | Kiambu, Kirinyaga, Nyeri, Nyandarua, Murang'a, Nairobi and Nakuru counties |
| Kipsigis | <https://youtu.be/6lsbITj7Nto> | Bomet, Kericho |
| Luhya-Idakho | <https://youtu.be/hyuCv9FPf0U> | Kakamega |
| Luhya-Isukha | <https://youtu.be/kd9A9v2wkWU> | Kakamega |
| Luhya-Kabras | <https://youtu.be/aiMdKFk2vS4> | Kakamega |
| Luhya-Khayo | <https://youtu.be/KaYGIQze69Y> | Busia |
| Luhya-Kinyala | <https://youtu.be/t4HO5KVHU9A> | Kakamega county |
| Luhya-Kisa | <https://youtu.be/EFRyfVTN__M> | Kakamega county |
| Luhya-Marachi | <https://youtu.be/jk-3VTkGKiE> | Busia and Kakamega counties |
| Luhya-Maragoli | <https://youtu.be/ietzo444zdM> | Transnzoia, Vihiga |
| Luhya-Marama | <https://youtu.be/mxhA3rGQaos> | Kakamega |
| Luhya-Samia | <https://youtu.be/x84ud8TK2yc> | Busia |
| Luhya-Tsotso | <https://youtu.be/_xiRrEOfxYw> | Kakamega county |
| Luhya-Wanga | <https://youtu.be/XFq0sWwWaA4> | Kakamega |
| Luo | <https://youtu.be/1ArrjQzM9uc> | Bomet, Homabay, Kisumu, Migori, Nyamira, Siaya |
| Maasai | <https://youtu.be/_bLj3Nf6wg0> | Bomet |
| Meru | <https://youtu.be/E2N99tjuJPs> | Meru, Tharaka Nithi |
| Mijikenda-Jibana | <https://youtu.be/HmlfH9g63II> | Kilifi, Kwale counties |
| Mijikenda-Kauma | <https://youtu.be/wY4wOyiUgIs> | Kilifi county |
| Mijikenda-Kambe | <https://youtu.be/mdi0gwiLWKY> | Kilifi country |
| Nandi | <https://youtu.be/-uG7fYsx-lI> | Nandi, Transnzoia, Uasin Gichu |
| Pokomo | <https://youtu.be/usXVy3nkH78> | Country wide |
| Pokot | <https://youtu.be/mcBokEt1_CY> | Elgeyo Marakwet |
| Sabaot | <https://youtu.be/pd8yy_Xl4uU> | Bungoma, Transnzoia |
| Somali | <https://youtu.be/ApKIDITvGso> | Marsabit, Garissa, Mandera, Isiolo counties |
| Swahili | <https://youtu.be/B2cnDwW-eb8> | Country wide |
| Swahili | <https://youtu.be/B2cnDwW-eb8> | Kenya ZOI |
| Taveta-Taita | <https://youtu.be/DUA4RhjmxhU> | Taita Taveta |

Kenya ZOI represents the following locations in Kenya: Bomet, Bungoma, Busia, Elgeyo Marakwet, Homabay, Kakamega, Kericho, Kisii, Kisumu, Kitui, Machakos, Makueni, Meru, Migori, Nandi, Nyamira, Siaya, Taita Taveta, Tharaka Nithi, Transnzoia, Uasin Gichu, and Vihiga.
