# Supplementary material for: Agricultural education in Africa using YouTube multilingual animations: A retrospective feasibility study assessing costs to reach language-diverse populations
Source: PLoS One. 2024 Apr 18;19(4):e0302136. doi: 10.1371/journal.pone.0302136 (PMC11025858; doi:10.1371/journal.pone.0302136)
Supplement: S2 File — YouTube data used to generate the language-specific models. (DOCX) [file pone.0302136.s002.docx]

**S2 Data link**

All YouTube data used to generate the language-specific models are available from the Purdue University Research Repository (PURR) database (DOI: 10.4231/TJK7-YD07). PURR is an online, collaborative working space and data-sharing platform to support Purdue researchers and their collaborators.
