# Supplementary material for: Agricultural education in Africa using YouTube multilingual animations: A retrospective feasibility study assessing costs to reach language-diverse populations
Source: PLoS One. 2024 Apr 18;19(4):e0302136. doi: 10.1371/journal.pone.0302136 (PMC11025858; doi:10.1371/journal.pone.0302136)
Supplement: S3 File — Model details for language-specific model generated from YouTube data for daily campaigns in Ghana for the language English (zone of influence) and considering number of viewers that watched 75% of the video. (DOCX) [file pone.0302136.s003.docx]

**S3 Modeling example**

Using YouTube data for daily campaigns in Ghana for the language English (zone of influence) and considering number of viewers that watched 75% of the video, one can notice the time series data for money spent and views are not stationary (see Figure S3.1-A and S3.1-C, respectively), and both variables present strong positive autocorrelation for at least eight consecutive days (see Figure S3.1-B and S3.1-D, respectively). Finally, one can see a positive correlation between daily money spent and the number of persons that watched 75% of the video (Figure S3.1-E).


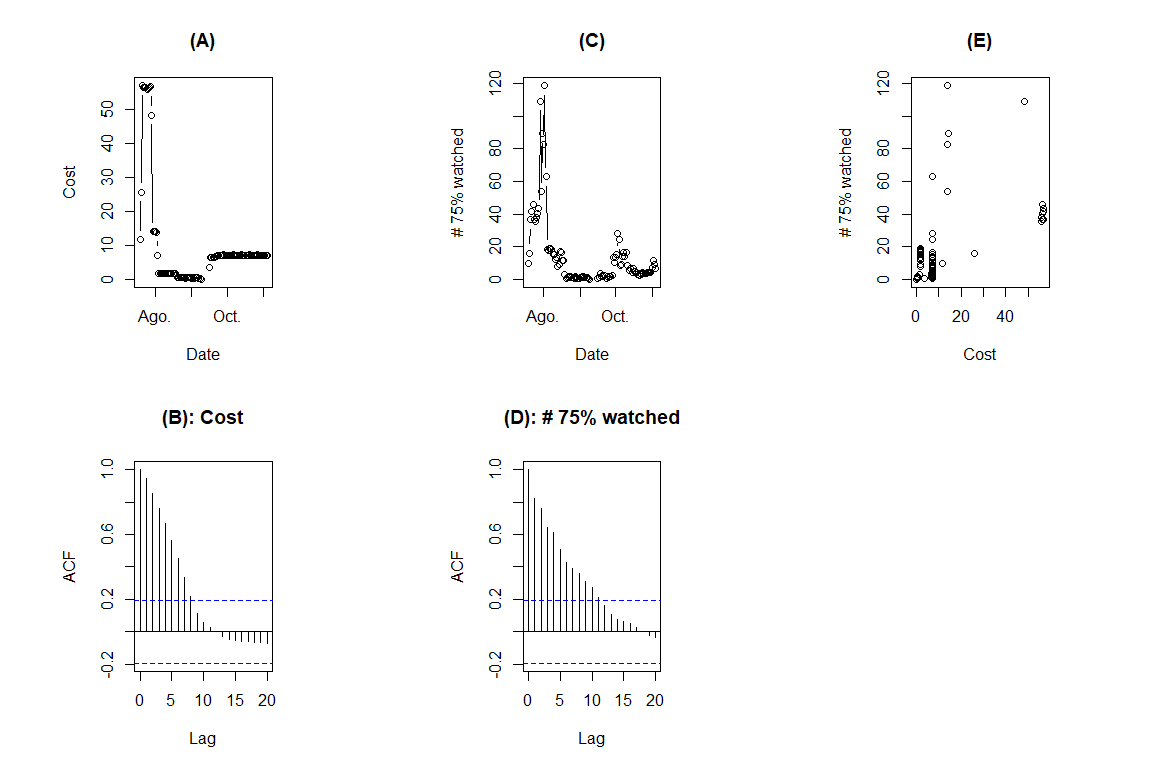


*Figure S3.1: Trajectory of cost (A) and number of viewers who watched 75% of video (C) over time and estimated autocorrelation for each variable (B,D), along with a scatterplot for cost and viewers (E) for the language-specific model English zone of influence in Ghana.*

The initial model explored in the study represented a simple linear regression model that estimated the effect of daily money spent ($X_{(t)})$on the expected number of daily viewers ($Y_{(t)}$).

$Y_{(t)}=\beta_{1}X_{(t)}+\epsilon_{(t)}$ [eq1]

The Durbin-Watson test was applied to the estimated time series error, $\epsilon_{(t)}$, to assess evidence or not of autocorrelation amongst daily residual error. Most language-specific models showed evidence of strong autocorrelation (i.e., observations amongst days are dependent) among the error estimates (see Figure S3.2 for example). To address the violation, one but most often two additional terms were added to the model representing the effects of the previous day number of viewers ($Y_{(t-1)}$) and/or previous day money spent ($X_{(t-1)}$) on the current day number of viewers ($Y_{(t)}$).

$Y_{(t)}=\beta_{1}Y_{(t-1)}+\beta_{2}X_{(t)}+\beta_{3}X_{(t-1)}+\epsilon_{(t)}$ [eq2]

When these terms were added, for most models, the observation independence assumption was no longer violated (see Figure S3.3).


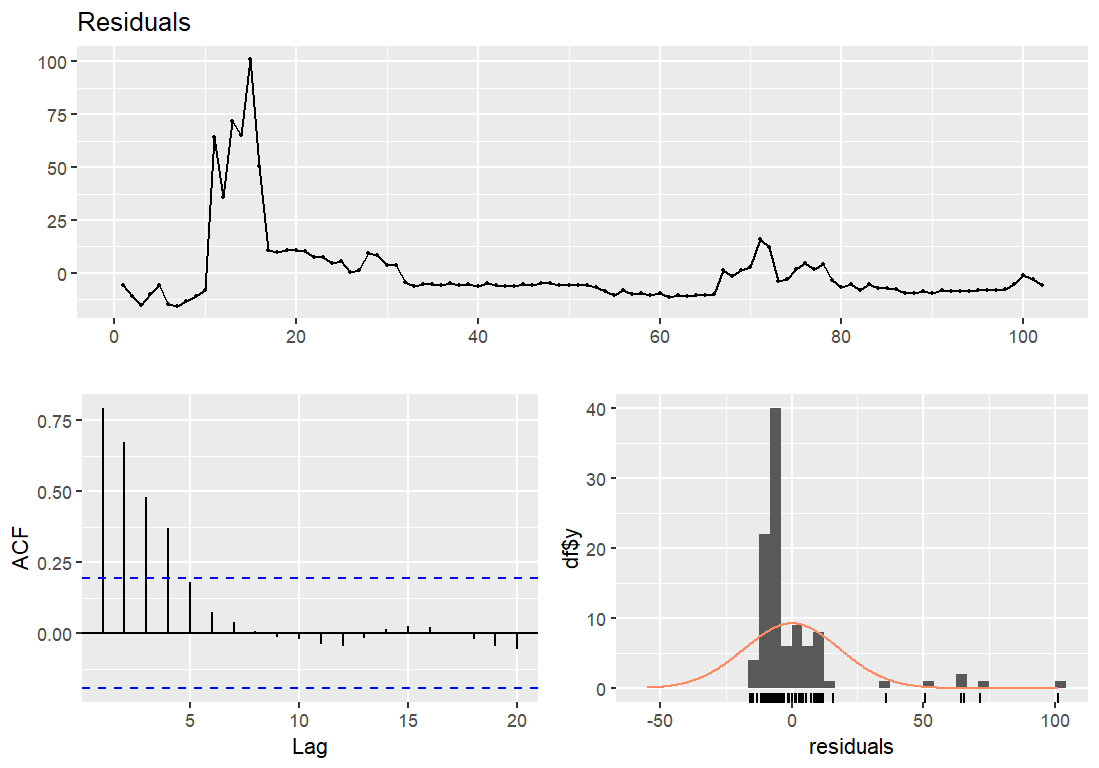


*Figure S3.2 Trajectory in residuals (*$\epsilon_{(t)})$*over time (A), estimated autocorrelation (B), and distribution of the estimated residuals (C) for the simple linear regression model (eq1) for the language-specific model English zone of influence in Ghana. Note that the correlation between days exceeds the threshold (i.e., blue dotted line) indicating the assumption of observation independence was violated.*


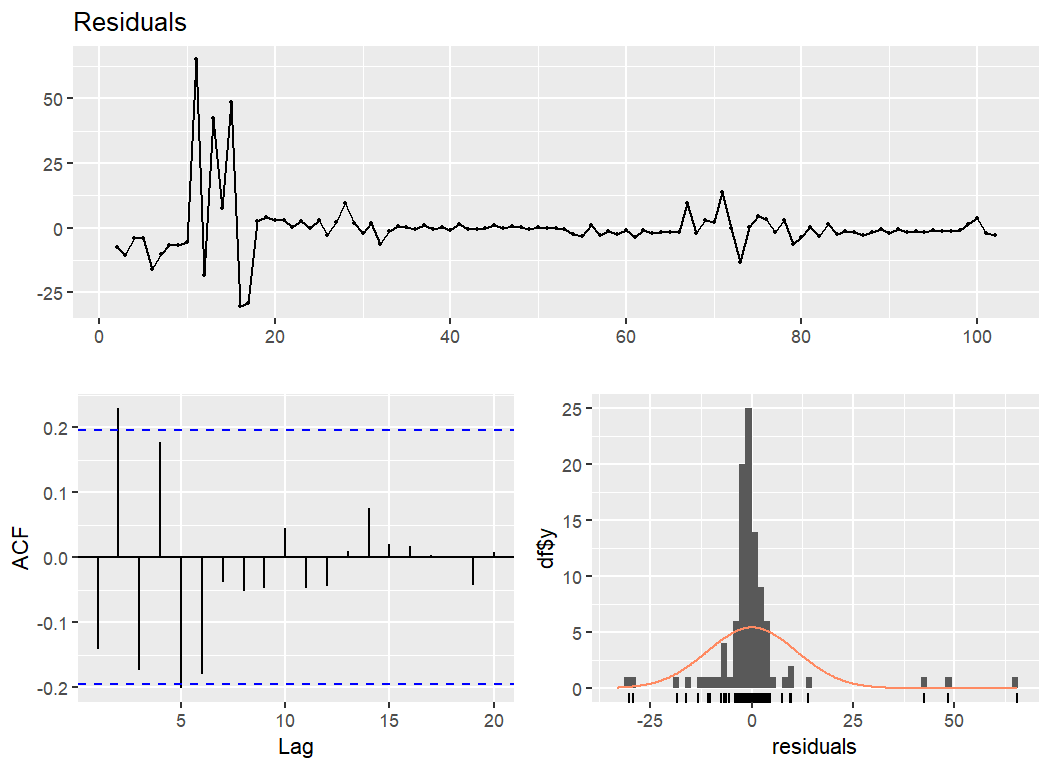


*Figure S3.3: Trajectory in residuals (*$\epsilon_{(t)})$*over time (A), estimated autocorrelation (B), and distribution of the estimated residuals (C) for the prespecified lag lineal regression model (eq2) for the language-specific model English ZOI in Ghana. Note the significant reduction in the estimated correlation between days.*

Furthermore, model [eq2] is compared with simpler models using the AIC statistics. For our example, Table S3.1 shows that the English (zone of influence) language model in Ghana that included previous number of viewers, current cost, and previous cost provides a better fit of the data. Furthermore, the Durbin-Watson test showed no evidence of autocorrelation among the residuals (p-value = 0.168).

*Table S3.1: Model comparison for the language-specific model English zone of influence in Ghana.*

| **Model (Lineal predictor)** | **AIC statistic** |
| --- | --- |
| Current cost  $\beta_{2}X_{(t)}$ | 887.12 |
| Current cost + previous cost  $\beta_{2}X_{(t)}+\beta_{3}X_{(t-1)}$ | 876.27 |
| Previous number of viewers + current cost  $\beta_{1}Y_{(t-1)}+\beta_{2}X_{(t)}$ | 781.46 |
| Previous number of viewers + current cost + previous cost  $\beta_{1}Y_{(t-1)}+\beta_{2}X_{(t)}+\beta_{3}X_{(t-1)}$ | 778.85 |
