# Supplementary material for: Agricultural education in Africa using YouTube multilingual animations: A retrospective feasibility study assessing costs to reach language-diverse populations
Source: PLoS One. 2024 Apr 18;19(4):e0302136. doi: 10.1371/journal.pone.0302136 (PMC11025858; doi:10.1371/journal.pone.0302136)
Supplement: Attachment — Submitted filename: PONE-D-23-30519_Comments_Reviewer.pdf [file pone.0302136.s005.pdf]

REVIEWERS' COMMENTS: PONE-D-23-30519

1. The study presents the results of original research.
Yes, the research results are original.

2. Results reported have not been published elsewhere.
The reviewer could not find the results in other publications. However, that may not sufficient because the reviewer's role was limited in this regard. Therefore, the authors should declare to the Journal that the results have not been published elsewhere.

3. Experiments, statistics, and other analyses are performed to a high technical standard and are described in sufficient detail.
The research methods and statistical analyses meet the requirements for a research manuscript.

4. Conclusions are presented in an appropriate fashion and are supported by the data.
The conclusions are based on the research findings. However, they should be separated from the discussions.

5. The article is presented in an intelligible fashion and is written in standard English.
The article is written in standard English.

6. The research meets all applicable standards for the ethics of experimentation and research integrity.
Yes, the research meets all applicable standards for the ethics of experimentation and research integrity.

7. The article adheres to appropriate reporting guidelines and community standards for data availability.

The primary data was made available by the authors.

DETAILED REVIEWER'S COMMENTS

1. Abstract

Add the summary of the recommendations in the abstract. Food insecurity should be removed from key words because it was not covered by the study.

2. Introduction

The introduction is well written with flow of ideas. Again, the research problem is clearly articulated. The following improvements should be made in the introduction.

- Provide the information for farmer to extension ration for countries that participated in the study (Ghana, Kenya and Nigeria) to supplement the ratio for sub-Saharan Africa. Again, indicate the reasons why the farmer to extension ratio is a high in sub-Saharan Africa or selected countries.
- Agricultural extension advisory services should be changed to agricultural extension and advisory services.
- At the end of the problem statement after indicating that the cost of YouTube and campaigns vary based on audience targeting, bidding, and ad format, there is a need to reflect on the studies that investigated the use of ICT in farming so that the knowledge gap can be clearly identified. By so doing, it will show the difference between the current study and other studies that investigated the use of ICT by farmers. Did other studies translate the YouTube videos to the selected languages in the study.

3. Methodology

Online education intervention

- The videos in figure 1 was not accessible. Therefore, the readers of the article will not be able to watch the videos. The content of the video should be provided in writing and/or the video link should be included the published manuscript.

Statistical analysis

Descriptive statistics such as median, range and IQR should be mentioned because they were also used to analyse data as shows by the results in Table 2. In most cases, media and IQR are when data is not normally distributed. If that was the case, then the results of the methods used to test data normality should be presented. If data was normally distributed, then the arithmetic mean, standard deviation and standard error of mean should be considered.

4. Results

The results are well presented.

5. Discussion

The discussion is well written; however, some of the information is about conclusions. Therefore, there is a need to separate discussions of the results from conclusions. Because of that, the new section about conclusions and recommendations should be added in the manuscript.
