# Supplementary material for: Agricultural education in Africa using YouTube multilingual animations: A retrospective feasibility study assessing costs to reach language-diverse populations
Source: PLoS One. 2024 Apr 18;19(4):e0302136. doi: 10.1371/journal.pone.0302136 (PMC11025858; doi:10.1371/journal.pone.0302136)
Supplement: Attachment — Submitted filename: Reviewer Response pr1.docx [file pone.0302136.s004.docx]

**PlosOne**

**Point-by-Point Response to Reviewers**

PONE-D-23-30519

Language-diverse agricultural education in Africa using YouTube Ad campaigns: A retrospective feasibility study

**Responses to Editor's Comments:**

Dear Dr. Reeves,

We've checked your submission and before we can proceed, we need you to address the following issues:

It has come to our attention that the manuscript seems to be a resubmission of the manuscript “PONE-D-23-13324”, which was previously considered by the journal.

It is a requirement by the journal that any manuscript resubmitted after a previous rejection is accompanied by a rebuttal letter outlining the response to the original reviewers' comments, so that the editors can establish whether the original concerns have been addressed. Can you therefore please provide us with a point-by-point response to the comments provided by the original reviewers, outlining the revisions carried out since the original submission.

As pointed out in the current submission, the original submission was pulled during the review process after a mistake was discovered in the analysis. The previous submission never completed the review process and we never received feedback from reviewers.  We mentioned the prior submission so that it would facilitate the review of the current submission by possibly having the same editor and reviewers.  There is no ability to reply to reviewer comments’ because no reviewers’ comment were provided for the original manuscript submission.  I hope this clarifies the issue.

The submitted manuscript has changes related to results presented; however the new results do not change the overall message of the paper.

Please see the track changes document for the updates to the revised manuscript.

Thank you for considering our manuscript!
