# Supplementary material for: Agricultural education in Africa using YouTube multilingual animations: A retrospective feasibility study assessing costs to reach language-diverse populations
Source: PLoS One. 2024 Apr 18;19(4):e0302136. doi: 10.1371/journal.pone.0302136 (PMC11025858; doi:10.1371/journal.pone.0302136)
Supplement: Attachment — Submitted filename: Response to reviewers pr9_gc1.docx [file pone.0302136.s006.docx]

**PlosOne**

**Point-by-Point Response**

**PONE-D-23-30519**

Agricultural education in Africa using YouTube multilingual animations: A retrospective feasibility study assessing costs to reach language-diverse populations

Dear editor and reviewers,

We express our gratitude for your insightful comments. Based on your feedback, we have implemented revisions to the paper, which are detailed in the point-by-point response below. Our comments are in *italics*. Parts taken from the paper are in *"quotation marks"*, and new sections added are *"underlined"*.

**Responses to Editor's Comments:**

Please include the following items when submitting your revised manuscript:

- A rebuttal letter that responds to each point raised by the academic editor and reviewer(s). You should upload this letter as a separate file labeled 'Response to Reviewers'.

*A Response to Reviewers document was uploaded as a separate file.*

- A marked-up copy of your manuscript that highlights changes made to the original version. You should upload this as a separate file labeled 'Revised Manuscript with Track Changes'.

*A Revised Manuscript with Track Changes document was uploaded as a separate file.*

- An unmarked version of your revised paper without tracked changes. You should upload this as a separate file labeled 'Manuscript'.

*A Manuscript document was uploaded as a separate file.*

**Journal Requirements:**

When submitting your revision, we need you to address these additional requirements.

1. Please ensure that your manuscript meets PLOS ONE's style requirements, including those for file naming. The PLOS ONE style templates can be found at

<https://journals.plos.org/plosone/s/file?id=wjVg/PLOSOne_formatting_sample_main_body.pdf> and

<https://journals.plos.org/plosone/s/file?id=ba62/PLOSOne_formatting_sample_title_authors_affiliations.pdf>

*The manuscript was modified to meet PLOS ONE's style requirements.*

2. Note from Emily Chenette, Editor in Chief of PLOS ONE, and Iain Hrynaszkiewicz, Director of Open Research Solutions at PLOS: Did you know that depositing data in a repository is associated with up to a 25% citation advantage (<https://doi.org/10.1371/journal.pone.0230416>)? If you’ve not already done so, consider depositing your raw data in a repository to ensure your work is read, appreciated and cited by the largest possible audience. You’ll also earn an Accessible Data icon on your published paper if you deposit your data in any participating repository (<https://plos.org/open-science/open-data/#accessible-data>).

*The data is stored on PURR, which is publicly available data repository. A link to this repository is provided in the Supplemental information (S2_File).*

3. Thank you for stating the following financial disclosure:

“This work was made possible by the generous support of the American people through the United States Agency for International Development (USAID,

<https://www.usaid.gov/>), under the terms of Contract No. 7200AA20LA00002 (Awardee: Purdue University; PI: BRP). USAID administers the U.S. foreign assistance

program providing economic and humanitarian assistance in more than 80 countries worldwide. The contents are the responsibility of the authors and do not necessarily reflect the views of USAID or the United States Government.”

Please state what role the funders took in the study.  If the funders had no role, please state: "The funders had no role in study design, data collection and analysis, decision to publish, or preparation of the manuscript."

If this statement is not correct you must amend it as needed.

Please include this amended Role of Funder statement in your cover letter; we will change the online submission form on your behalf.

*We add the following statement to the cover letter.*

*"The funders had no role in study design, data collection and analysis, decision to publish, or preparation of the manuscript."*

4. Thank you for stating the following in the Competing Interests section:

“N. Peter Reeves is the Founder and President of Sumaq Life LLC. Sumaq Life LLC applies mathematical modeling approaches to understand complex systems to optimize their performance. It receives funding for these services, including work on the current project. The remaining authors declare no conflicts of interest in the production of this work.”

Please confirm that this does not alter your adherence to all PLOS ONE policies on sharing data and materials, by including the following statement: "This does not alter our adherence to  PLOS ONE policies on sharing data and materials.” (as detailed online in our guide for authors <http://journals.plos.org/plosone/s/competing-interests>).  If there are restrictions on sharing of data and/or materials, please state these. Please note that we cannot proceed with consideration of your article until this information has been declared.

Please include your updated Competing Interests statement in your cover letter; we will change the online submission form on your behalf.

*We add the following statement to the cover letter.*

*“N. Peter Reeves is the Founder and President of Sumaq Life LLC. Sumaq Life LLC applies mathematical modeling approaches to understand complex systems to optimize their performance. It receives funding for these services, including work on the current project. This does not alter our adherence to  PLOS ONE policies on sharing data and materials. The remaining authors declare no conflicts of interest in the production of this work.”*

5. When completing the data availability statement of the submission form, you indicated that you will make your data available on acceptance. We strongly recommend all authors decide on a data sharing plan before acceptance, as the process can be lengthy and hold up publication timelines. Please note that, though access restrictions are acceptable now, your entire data will need to be made freely accessible if your manuscript is accepted for publication. This policy applies to all data except where public deposition would breach compliance with the protocol approved by your research ethics board. If you are unable to adhere to our open data policy, please kindly revise your statement to explain your reasoning and we will seek the editor's input on an exemption. Please be assured that, once you have provided your new statement, the assessment of your exemption will not hold up the peer review process.

*The data is stored on PURR, which is publicly available data repository. A link to this repository is provided in the Supplemental information (S2_File).*

6. We note that Figure 1 in your submission contain copyrighted images. All PLOS content is published under the Creative Commons Attribution License (CC BY 4.0), which means that the manuscript, images, and Supporting Information files will be freely available online, and any third party is permitted to access, download, copy, distribute, and use these materials in any way, even commercially, with proper attribution. For more information, see our copyright guidelines: <http://journals.plos.org/plosone/s/licenses-and-copyright>.

We require you to either (1) present written permission from the copyright holder to publish these figures specifically under the CC BY 4.0 license, or (2) remove the figures from your submission:

a. You may seek permission from the original copyright holder of Figure 1 to publish the content specifically under the CC BY 4.0 license.

We recommend that you contact the original copyright holder with the Content Permission Form (<http://journals.plos.org/plosone/s/file?id=7c09/content-permission-form.pdf>) and the following text:

“I request permission for the open-access journal PLOS ONE to publish XXX under the Creative Commons Attribution License (CCAL) CC BY 4.0 (<http://creativecommons.org/licenses/by/4.0/>). Please be aware that this license allows unrestricted use and distribution, even commercially, by third parties. Please reply and provide explicit written permission to publish XXX under a CC BY license and complete the attached form.”

Please upload the completed Content Permission Form or other proof of granted permissions as an "Other" file with your submission.

In the figure caption of the copyrighted figure, please include the following text: “Reprinted from [ref] under a CC BY license, with permission from [name of publisher], original copyright [original copyright year].”

b. If you are unable to obtain permission from the original copyright holder to publish these figures under the CC BY 4.0 license or if the copyright holder’s requirements are incompatible with the CC BY 4.0 license, please either i) remove the figure or ii) supply a replacement figure that complies with the CC BY 4.0 license. Please check copyright information on all replacement figures and update the figure caption with source information. If applicable, please specify in the figure caption text when a figure is similar but not identical to the original image and is therefore for illustrative purposes only.

*Figure 1 was removed. A link to the YouTube video was provided in the manuscript.*

*"A computer-animated YouTube video was created to educate farmers on how to safely store beans using readily available, sanitized, sealable containers such as jerrycans to avoid post-harvest loss (see the following link to access video https://rapid.sawbo-animations.org/video/1445)."*

7. Please include captions for your Supporting Information files at the end of your manuscript, and update any in-text citations to match accordingly. Please see our Supporting Information guidelines for more information: <http://journals.plos.org/plosone/s/supporting-information>.

*We have included captions for Supporting Information files (S1_File.pdf, S2_File.pdf, S3_File.pdf) at the end of the manuscript.*

8. Please review your reference list to ensure that it is complete and correct. If you have cited papers that have been retracted, please include the rationale for doing so in the manuscript text, or remove these references and replace them with relevant current references. Any changes to the reference list should be mentioned in the rebuttal letter that accompanies your revised manuscript. If you need to cite a retracted article, indicate the article’s retracted status in the References list and also include a citation and full reference for the retraction notice.

*The reference list has been checked to ensure it is complete and correct.*

*The following changes to the reference list were made.*

*The Speranza reference was removed*

*9	Speranza, C. I., Ochege, F. U., Nzeadible, T. C. & Agwu, A. E. in Beyond Agricultural Impacts	(ed Theobald Frank Theodory Nkulumo Zinyengere, Million Gebreyes, Chinwe Ifejika Speranza) Ch. 12, 241-274 (Academic Press, 2018).*

*"The average ratio of agricultural extension agents to farmers averages around 1 to 5,000 in Sub-Saharan Africa, with some areas having ratios as high as 1 to 22,000(9)."*

*The above reference was replaced with country specific estimates of extension agents to farmer ratios.*

*9.	McNamara P, Dale J, Keane J, Ferguson O. Strengthening pluralistic agricultural extension in Ghana. Modernizing extension and advisory services discussion paper, USAID. 2014.*

*10.	Odongo D. Agricultural Information Access Among Smallholder Farmers: Comparative Assessment of Peri-Urban and Rural Settings in Kenya. Agricultural Information Worldwide. 2013/2014;6:133-7.*

*11.	Davis K, Lion K, Arokoyo T. Organisational Capacities And Management Of Agricultural Extension Services In Nigeria: Current Status. South African Journal of Agricultural Extension (SAJAE). 2019;47(2).*

*"The ratio of agricultural extension agents to farmers are notably high, with estimates revealing ratios of 1 to 1,300 in Ghana(**9**), 1 to 1,100 in Kenya(**10**), and even lower ratios in Nigeria, ranging from 1 to 5,000 to as high as 1 to 10,000(**11**). This disparity may, in part, stem from organizational and managerial challenges within extension services, ambiguous extension mandates, and inadequate remuneration for personnel(**12**)."*

*Also, the Samson Olayemi reference was added to explain this disparity.*

*12.	Samson Olayemi S, Taiye Oduntan F. Effectiveness of Traditional Extension Models among Rural Dwellers in Sub-Saharan African Communities. International Journal of Advanced Research. 2020;08(04):401-15.*

*Finally, the Moyo reference was added to the reference list. The study was cited but it was missing in the prior submission reference list.*

*34.	Moyo S. Family farming in sub-Saharan Africa: its contribution to agriculture, food security and rural development. 2016. No. 15 Working paper.*

*"However, in many African countries, the divide between urban and rural is not well defined, with urban populations also owning and maintaining farms(32, 33) and communication between family members and clans from urban to rural areas being more fluid than in more developed Western economies(34)."*

**Responses to Reviewers Comments**

**Reviewer #1:**

*Thank you for your thoughtful feedback and providing time to reviewing the manuscript.*

1. The study presents the results of original research.
Yes, the research results are original.

*No action required.*


2. Results reported have not been published elsewhere.
The reviewer could not find the results in other publications. However, that may not sufficient because the reviewer’s role was limited in this regard. Therefore, the authors should declare to the Journal that the results have not been published elsewhere.

*The authors declare to the Journal that the results have not been published elsewhere in the cover letter.*

*"This manuscript is original and has not been previously published, including on preprint servers, nor is it being considered elsewhere until a decision is made as to its acceptability by the PLOS ONE Editorial Review Board."*

3. Experiments, statistics, and other analyses are performed to a high technical standard and are described in sufficient detail.
The research methods and statistical analyses meet the requirements for a research manuscript.

*No action required.*

4. Conclusions are presented in an appropriate fashion and are supported by the data.
The conclusions are based on the research findings. However, they should be separated from the discussions.

*A Conclusions section has been added. Conclusions are separated from the Results and discussion section.*

5. The article is presented in an intelligible fashion and is written in standard English.
The article is written in standard English.

*No action required.*

6. The research meets all applicable standards for the ethics of experimentation and research
integrity.
Yes, the research meets all applicable standards for the ethics of experimentation and research integrity.

*No action required.*

7. The article adheres to appropriate reporting guidelines and community standards for data
availability.
The primary data was made available by the authors.

*No action required.*

DETAILED REVIEWER’S COMMENTS

1. Abstract
Add the summary of the recommendations in the abstract. Food insecurity should be removed from key words because it was not covered by the study.

*A summary of the recommendations was added to the abstract.*

*"... Results also showed that many local language campaigns performed well, opening the possibility that targeted knowledge dissemination on topics of importance to local populations, is potentially cost effective. In addition, such targeted information dissemination appears feasible, even during regional and global crises when in-person training may not be possible. In summary, leveraging multilingual computer-animations and digital platforms such as YouTube shows promise for conducting large-scale agricultural education campaigns. The findings of the current study provides the justification to pursue a more rigorous prospective study to verify the efficacy of knowledge exchange and societal impact through this form of information dissemination channel. "*

*Food insecurity was removed from keywords.*

2. Introduction
The introduction is well written with flow of ideas. Again, the research problem is clearly articulated. The following improvements should be made in the introduction.
• Provide the information for farmer to extension ration for countries that participated in the study (Ghana, Kenya and Nigeria) to supplement the ratio for sub-Saharan Africa. Again, indicate the reasons why the farmer to extension ratio is a high in sub-Saharan Africa or selected countries.

*Country specific extension agent to farmer ratios were provided in the introduction along with potential reasons for the lack of extension agents.*

*"Given the strong link between agricultural productivity and a country's economic growth(4, 5) and population health(6), most governments invest significant funding into large-scale training programs to improve agricultural practices through agricultural extension and advisory services(7, 8). Unfortunately, resources for conducting such training programs are often limited, thus reducing their potential reach and impact. The ratio of agricultural extension agents to farmers are notably high, with estimates revealing ratios of 1 to 1,300 in Ghana(9), 1 to 1,100 in Kenya(10), and even higher ratios in Nigeria, ranging from 1 to 5,000 to as high as 1 to 10,000(11). This disparity may, in part, stem from organizational and managerial challenges within extension services, ambiguous extension mandates, and inadequate remuneration for personnel(12). To address resource constraints, new approaches should be considered for deploying agricultural training programs, to complement traditional in-person agricultural extension training events."*

• Agricultural extension advisory services should be changed to agricultural extension and advisory services.

*Agricultural extension advisory services was changed to agricultural extension and advisory services throughout the manuscript.*

• At the end of the problem statement after indicating that the cost of YouTube and campaigns vary based on audience targeting, bidding, and ad format, there is a need to reflect on the studies that investigated the use of ICT in farming so that the knowledge gap can be clearly identified. By so doing, it will show the difference between the current study and other studies that investigated the use of ICT by farmers. Did other studies translate the YouTube videos to the selected languages in the study.

*The other ICT studies also used linguistically and culturally localized computer-animations but they were not delivered through YouTube but instead through in-person training. In-person training events have the common problem of limited extension resources (e.g. high extension agent to farmer ratios), making scaling of these programs challenging. The following sentence was added to the introduction.*

*"One potential solution for overcoming literacy and language barriers is the use of information and communication technologies (ICTs) in the form of culturally and linguistically localized, computer-animated training videos, specifically developed for low-literacy, non-English speaking audiences(**17**,* *18**). Studies suggest such ICTs promote learning gains and adoption of technologies taught in the animations and lead to novel innovations in agricultural communities(**19-21**). These prior ICT studies using computer-animated training videos predominately relied on conventional in-person training events. It has been suggested that ICTs could increase access to agricultural extension and advisory services(**22-25**), thus complementing and enhancing existing extension training programs(**26**). However, despite the promise of ICTs, traditional in-person training encounters scalability challenges, primarily stemming from the high ratios of extension agents to farmers."*

3. Methodology
Online education intervention
• The videos in figure 1 was not accessible. Therefore, the readers of the article will not be able to watch the videos. The content of the video should be provided in writing and/or the video link should be included the published manuscript.

*The following link for the video was added to the manuscript.*

*"A computer-animated YouTube video was created to educate farmers on how to safely store beans using readily available, sanitized, sealable containers such as jerrycans to avoid post-harvest loss (see the following link to access video* [*https://rapid.sawbo-animations.org/video/1445*](https://rapid.sawbo-animations.org/video/1445)*). Viewers can download the video for free, allowing them to view the videos offline on their mobile device and share the videos with others. This offline usage and propagation of video sharing is not captured in the current study."*

Statistical analysis
Descriptive statistics such as median, range and IQR should be mentioned because they were also used to analyse data as shows by the results in Table 2. In most cases, media and IQR are when data is not normally distributed. If that was the case, then the results of the methods used to test data normality should be presented. If data was normally distributed, then the arithmetic mean, standard deviation and standard error of mean should be considered.

*Table 2 provides an overview of the main input and output variables through descriptive statistics. To derive confidence intervals to allow for inferences in the data, a bootstrapping technique was employed. Unlike traditional methods, bootstrapping does not necessitate data to adhere to a normal distribution, hence accommodating non-symmetrical confidence intervals, as illustrated in Fig 2-3. Because bootstrapping does not require normally distributed data, no tests for normality were conducted or presented in the manuscript. Nevertheless, we decided to present the mean and standard deviation of the main input and output variables in Table 2 to inform readers on the normality of the data.*

Table 2. Observed descriptive statistics reflecting the daily performance of YouTube ad campaigns in Ghana, Kenya, Nigeria, and combined.

|  |  | Ghana | Kenya | Nigeria | Combined |
| --- | --- | --- | --- | --- | --- |
| Daily money spent per campaign (USD) | Mean | $9.26 | $19.15 | $8.76 | $14.54 |
|  | Standard deviation | $15.97 | $37.26 | $15.84 | $29.97 |
|  | Median | $7.13 | $9.39 | $7.14 | $7.19 |
|  | Interquartile | $1.11 – $7.26 | $1.27 – $21.58 | $0.93 – $7.27 | $1.12 – $21.48 |
|  | Range | $0.02 – $159.90 | $0.01 - $446.30 | $0.02 – $158.16 | $0.01 – $446.30 |
| Daily number of viewers per campaign who watched 25% of video | Mean | 21.8 | 24 | 4.8 | 18.9 |
|  | Standard deviation | 84.9 | 51.6 | 15.5 | 56 |
|  | Median | 5.2 | 8.9 | 1.3 | 4.7 |
|  | Interquartile | 1.9 - 13.7 | 2.4 - 18.7 | 0.4 - 2.8 | 1.3 - 14.4 |
|  | Range | 0 - 1533.9 | 0 - 747.5 | 0 - 205.9 | 0 - 1533.9 |
| Daily number of viewers per campaign who watched 75% of video | Mean | 9.7 | 10.1 | 1.9 | 8.1 |
|  | Standard deviation | 38.9 | 23.1 | 6.4 | 25.3 |
|  | Median | 2.1 | 3.7 | 0.5 | 1.9 |
|  | Interquartile | 0.8 - 5.8 | 1.0 - 8.1 | 0.1 - 1.1 | 0.5 - 5.9 |
|  | Range | 0 - 747.2 | 0 - 376.0 | 0 - 89.9 | 0 - 747.2 |

4. Results
The results are well presented.

*No action required.*

5. Discussion
The discussion is well written; however, some of the information is about conclusions. Therefore, there is a need to separate discussions of the results from conclusions. Because of that, the new section about conclusions and recommendations should be added in the manuscript.

*The conclusions and recommendations were separated from the discussion and added to a new section referred to as Conclusions.*

***"****Conclusions*

*This feasibility study suggests that online information dissemination through YouTube ads can reach significant numbers of language, diverse viewers in Africa. By demonstrating feasibility, prospective intervention studies are warranted to confirm that videos reach their intended audience, viewing videos lead to adoption of techniques, and adoption of techniques translate to tangible societal benefits. If subsequent studies demonstrate impactful translation, it may be imperative for donor agencies to develop working relationships with online platforms like YouTube to develop “bulk” costing structures that may drive down these costs further. Such collaborations could enable these platforms to become effective information dissemination channels in international development, especially in times of crisis be they regional or global."*

**Reviewer #2:** 
The topic at hand is fascinating, innovative, and certainly worth investigating. With so much untapped agricultural potential in many African countries, this study presents a fantastic opportunity to reach farmers beyond traditional extension routes. However, I do have some doubts regarding the thoroughness of the authors' research. The linear correlation between YouTube ads and agricultural education knowledge uptake may be oversimplified without a pre-post experimental design. Additionally, while the primary objective of the study was to assess feasibility in terms of cost, I remain unconvinced that clicks and views alone indicate knowledge gains, which ultimately undermines the purpose of the spending. To address these concerns, I suggest incorporating a component of the research that measures knowledge uptake both before and after the intervention.

*Thank you for your encouraging feedback and for taking the time to review our manuscript.*

*We acknowledge the reviewer's concerns regarding the lack of specific evidence supporting knowledge gain through YouTube. While there is existing evidence indicating knowledge acquisition through computer-animated training videos (as referenced in the manuscript), the extent of such knowledge transfer via YouTube dissemination remains uncertain. The proposed pre-post experimental study suggested by the reviewer would undoubtedly demand substantial resources for completion and was not initially incorporated into the project due to the limited budget and stringent timelines aimed at addressing food insecurity in Africa during the Covid-19 pandemic.*

*To justify the need for the pre-post experimental study and to aid in its design, we conducted a retrospective study. This retrospective analysis offers valuable insights into the costs associated with reaching language-diverse populations. By demonstrating feasibility through this retrospective approach, we bolster the case for undertaking the more intricate and resource-intensive prospective study. Moreover, having the preliminary study published in a reputable peer-reviewed journal strengthens the argument for potential sponsors, demonstrating that the research endeavor merits further pursuit.*

*We made several changes to the manuscript to address some of the reviewer's concerns.*

*We slightly changed the title to clarify that this is a feasibility study to assess the ability of YouTube information dissemination to reach language-diverse viewers.*

*"Agricultural education in Africa using YouTube multilingual animations: A retrospective feasibility study assessing costs to reach language-diverse populations"*

*We added a statement to the end of the abstract to highlight that further research is needed to confirm knowledge gain.*

*"... Results also showed that many local language campaigns performed well, opening the possibility that targeted knowledge dissemination on topics of importance to local populations, is potentially cost effective. In addition, such targeted information dissemination appears feasible, even during regional and global crises when in-person training may not be possible. In summary, leveraging multilingual computer-animations and digital platforms such as YouTube shows promise for conducting large-scale agricultural education campaigns. The findings of the current study provides the justification to pursue a more rigorous prospective study to verify the efficacy of knowledge exchange and societal impact through this form of information dissemination channel."*

*Also, we moved up the limitation section to follow the main findings of the paper. We felt it was important to acknowledge limitation before discussing the implications of the findings.*

*Finally, we soften assertions that YouTube ad campaigns are cost-effective for information dissemination*

*"... targeted knowledge dissemination on topics of importance to local populations, is potentially cost effective."*

*"Online agricultural education using YouTube ads could be a cost-effective method for reaching large populations."*

*"However, further research is required to confirm that the intended audience was reached and that knowledge gain occurred in viewers."*
